# Autoimmunity, Infections, and the Risk of Monoclonal Gammopathy of Undetermined Significance

**DOI:** 10.3389/fimmu.2022.876271

**Published:** 2022-04-28

**Authors:** Aðalbjörg Ýr Sigurbergsdóttir, Thorvardur Jon Love, Sigurður Yngvi Kristinsson

**Affiliations:** ^1^Faculty of Medicine, University of Iceland, Reykjavik, Iceland; ^2^Department of Science and Research, Landspitali University Hospital, Reykjavik, Iceland; ^3^Department of Haematology, Landspitali University Hospital, Reykjavik, Iceland

**Keywords:** monoclonal gammopathy of undetermined significance, autoimmune diseases, infections, chronic antigen stimulation, iStopMM study, risk

## Abstract

Various epidemiological studies, including case reports and -series in addition to larger, population-based studies, have reported an increased prevalence of monoclonal gammopathy of undetermined significance (MGUS) and multiple myeloma in individuals with a prior history of immune-related conditions. This is believed to support the role of chronic antigen stimulation in the pathogenesis of these conditions. In this short review, we summarize some of the largest population-based studies researching autoimmune diseases, infections, and the subsequent risk of MGUS, and discuss our understanding on its etiology and pathogenesis. Furthermore, we highlight important methodological limitations of previous studies in the field, but almost all studies on MGUS have been based on clinical, possibly biased, cohorts. Finally, we discuss future directions in researching the associations of MGUS and other disorders, including immune-related conditions, where screening studies play an important role.

## Introduction

Monoclonal gammopathy of undetermined significance (MGUS) is an asymptomatic, pre-malignant plasma cell disorder, preceding multiple myeloma (MM) and related disorders, including Waldenström’s macroglobulinemia and amyloid light-chain (AL) amyloidosis (1, 2). MGUS is characterized by monoclonal immunoglobulins (M-protein) <3.0 g/dL, bone marrow plasma cell infiltration <10%, and the absence of hypercalcemia, renal insufficiency, anemia, or bone lesions (CRAB criteria). Furthermore, a free light chain (FLC) ratio between 0.26 and 1.65 and a urinary protein <500 mg/24 hours is required (**Table 1**) (10). MGUS is present in around 3-4% of the general population over the age of 50 and around 6% of those over the age of 70 (11, 12). All MM cases are preceded by MGUS (13, 14). However, only a small proportion of individuals with MGUS ever progresses to a malignant disease, with an annual risk of progression from MGUS to MM of around 1% (2).

**Table 1:** Autoimmune diseases associated with MGUS.

Autoimmune disease	Number of studies showing significant associations	Number of studies showing no associations
Total autoimmune diseases	3 (3–5)	–
Autoantibodies detectable	3 (3–5)	–
Systemic involvement	3 (3–5)	–
Rheumatoid arhritis	2 (3, 5)	1 (6)*
Systemic sclerosis	3 (3–5)	1 (6)*
Sjögren syndrome	3 (3, 7, 8)	2 (5, 6)*
Polymyositis/dermatomyositis	2 (5, 8)	3 (3, 4, 6)*
Kikuchi disease	1 (7)	–
Organ involvement	3 (3–5)	–
Discoid lupus erythematous	1 (9)	2 (3, 6)*
Neuromyelitis optica	1 (7)	–
Autoimmune thrombocytopenia	2 (3, 8)	–
Autoimmune hemolytic anemia	1 (4)	1 (3)
Pernicious anemia	3 (3–5)	1 (6)*
Guillian-Barré syndrome	1 (3)	–
Celiac disease	1 (3)	–
Chronic rheumatoid heart disease	1 (3)	–
Autoantibodies not detectable	2 (3, 4)	1 (5)
Ankylosing spondylitis	4 (3–5, 7)	1 (6)*
Vitiligo	1 (8)	–
Polymyalgia rheumatica	1 (3)	–
Giant cell arteritis	1 (3)	–
Aplastic anemia	1 (3)	–

–indicates not applicable.

*indicates that the study is based on a screened population.

An intermediate stage between MGUS and MM is smoldering multiple myeloma (SMM), characterized by a serum M-protein ≥3.0 g/dL or a urinary M-protein ≥500 mg per 24 hours and/or a clonal bone marrow plasma cell infiltration of 10-60%, in the absence of end-organ damage (CRAB) or an involved:uninvolved serum FLC ratio of ≥100 or ≤0.01, and with ≤1 focal lesions on magnetic resonance imaging (MRI) (10). In SMM, the annual risk of progression to malignancy is 10% in the first 5 years, 3% in the following 5 years and 1% after that (15).

Predicting which MGUS patients will progress to malignancy has proved challenging. Current risk stratification models stratify MGUS-patients into four risk categories based on three risk factors: serum M-protein concentration ≥15 g/L, non-IgG isotype, and an FLC ratio <0.26 or >1.65. The presence of all three risk factors is considered high-risk MGUS, with a 58% combined 20-year risk of progression, without death accounted for as a competing risk. Conversely, absence of all risk factors is considered low-risk MGUS, with a 5% 20-year risk of progression (16).

In MGUS, as well as MM, the M-protein is secreted by post-germinal center plasma cells that have undergone somatic hypermutation, antigen selection, and immunoglobulin heavy-chain (IgH) class-switch recombination (17, 18). Various known genetic factors distinguish clonal plasma cells in MGUS, SMM and MM from healthy plasma cells; however, distinguishing the clonal plasma cells in MGUS from those in MM has proven more difficult. Overexpression of certain miRNA expression patterns have been detected by MM cell lines and tumors, but not in MGUS; however, more analyses are needed before these patterns can be effectively used to distinguish MGUS from MM tumors (18). Additionally, some shared cytogenic abnormalities (CAs) are found in MGUS and MM, including IgH-translocations, aneuploidy, and chromosome 13q deletion, but how they affect the development of MGUS has not been established (18). Nevertheless, certain CAs have been linked with higher risk of progression compared with others, especially regarding the progression from SMM to MM (19). Additional events, such as K-RAS activation and increased MYC expression or dysregulation, may affect the transition to MM, but further studies are required to determine their roles in the progression to malignancy (18).

The etiology of MGUS is largely unknown. However, several risk factors for the disorder have been identified, including male gender and increasing age (11). Racial disparities in MGUS prevalence have also been described by several studies (20–23). Landgren et al. reported a 1.97-fold higher prevalence of MGUS in black men from Ghana compared with white men from Minnesota, in their study on 917 Ghanaian- and 7,996 white men (22). Furthermore, in a study on 4 million black and white US veterans, the prevalence of MGUS was 3.0-fold higher in blacks. Interestingly, the subsequent risk of MM during the first 10 years of follow-up was similar between blacks and whites (p = 0.37), suggesting a similar precursor condition in those of European and African descent (21). In contrast, the prevalence of MGUS appears to be lower in Asian populations, as reported in a study of 52,802 Japanese individuals (23).

Population-based studies on Swedish subjects have identified an increased risk of MGUS among first-degree relatives of MGUS-, MM, and WM/lymphoplasmacytic lymphoma (LPL)-patients (24–26). Among 4,458 MGUS patients, 17,505 population-based controls, and first-degree relatives of patients and controls a 2.8-fold risk of MGUS was observed in first-degree relatives of MGUS-patients, in addition to an increased risk of MM (RR 2.9), WM/LPL (RR 4.0), and chronic lymphocytic leukemia (CLL, RR 2.0), compared with relatives of controls (24). An increased risk of MGUS was also observed in first-degree relatives of MM-patients (RR 2.1), compared with relatives of controls, in a study of 13,896 MM patients, 37,838 first-degree relatives and matched controls (25). Finally, first-degree relatives of LPL/WM patients had a 5.0-fold risk of MGUS in a study on 2,144 LPL/WM patients, 8.279 matched controls and first-degree relatives (26). These studies, in addition to racial disparities in the prevalence of MGUS, suggest genetic predisposition to MGUS and its consequent malignant forms. Recent genome wide association studies (GWAS) have identified several single nucleotide polymorphisms (SNPs) associated with MGUS, MM or both conditions, further indicating genetic roles in the etiology of MGUS and MM (27).

Several environmental factors have been identified as risk factors for the development of MGUS. Iwanaga et al. reported a higher prevalence of MGUS among individuals exposed to radiation at a young age in a study of >50,000 survivors of the atomic bomb in Nagasaki in 1945 (28). Exposure to pesticides has also been associated with an increased risk of MGUS, as reported in a study where 678 male pesticide applicators were compared with the 9,469 men representative of the general population (OR 1.9). A higher prevalence of MGUS was also specifically associated with an exposure to the chlorinated insecticide dieldrin (OR 5.6), the fumigant mixture carbon-tetrachloride/carbon disulfide (OR 3.9), and the fungicide chlorothalonil (OR 2.4) (29). Higher MGUS prevalence has also been reported in US veterans exposed to the herbicide Agent Orange (OR 2.37), used by the US Air Force in Vietnam from 1962-1971 (30).

In recent years, emerging evidence supporting the role of immune-related factors in the pathogenesis of MGUS and its progression to MM has been reported, suggesting that autoimmune diseases, infections, and inflammatory conditions stimulate a chronic antigen response, possibly triggering the development of MGUS (3–5). In this review, we will provide an overview of the current literature with regards to autoimmune diseases, infections, and the subsequent risk of MGUS. Furthermore, we will discuss important methodological issues in past studies and the importance of novel screening studies for further evaluation of disease associations with MGUS.

## MGUS and Autoimmune Diseases

Immune dysregulation is believed to play a major role in lymphomagenesis (31). However, the association between immune-related conditions and the risk of plasma cell disorders is less well-established (32). Numerous studies have linked MGUS and MM with various autoimmune disorders. Several case reports and -series have described detection of monoclonal gammopathies in patients previously, or coincidentally, diagnosed with various autoimmune conditions; including discoid lupus erythematous, Sjögren’s syndrome, neuromyelitis optica, Kikuchi disease, ankylosing spondylitis, autoimmune thrombocytopenia, polymyositis, and vitiligo (**Table 1**) (7–9). These studies suggest that chronic antigen stimulation can act as a trigger for plasma cell dyscrasias, a theory first presented in 1964 (33). Alternatively, they may suggest an increased susceptibility to immune-related conditions among MGUS-patients.

A few cohort studies have also studied autoimmune diseases and subsequent risk of MGUS. A retrospective cohort study of 2,046 male US veterans with MGUS and 4,641 with MM identified a significantly increased risk of MGUS in individuals with a prior autoimmune disease (RR 1.67, 95% CI 1.47-1.90), particularly of those with detectable autoantibodies (RR 1.78, 95% CI 1.54-2.06). The risk was similarly elevated for diseases with systemic- and organ involvement (RR 1.95 and 1.62, 95% CIs 1.57-2.42 and 1.35-1.94) compared with non-MGUS controls. Several specific autoimmune conditions were also associated with an increased risk of MGUS, including systemic sclerosis, autoimmune hemolytic anemia, and ankylosing spondylitis. An increased risk of MM was also reported in association with previously diagnosed autoimmune diseases, both in total and for specific disorders (**Table 1**). However, the RRs were generally lower for MM than for MGUS (4). These finding suggest that at least some of the abovementioned autoimmune diseases might activate the development of MGUS and MM. Alternatively, they might represent a bias due to increased testing for MGUS in patients with autoimmune disease (4).

In a large, population-based, retrospective cohort study from Sweden, on 5,403 MGUS and 19,112 MM cases, 96,617 matched controls and 262,931 first-degree relatives, a personal history of autoimmune diseases increased the risk of MGUS (OR 2.1, 95% CI 1.9-2.4). Furthermore, a personal history of rheumatoid arthritis (RA), systemic sclerosis, Sjögren syndrome, pernicious anemia, immune thrombocytopenia, Guillain-Barré syndrome, celiac disease, chronic rheumatic heart disease, ankylosing spondylitis, polymyalgia rheumatica, giant cell arteritis, or aplastic anemia was associated with an increased risk of MGUS, suggesting that autoimmunity or the treatment of autoimmune diseases increases the risk of developing MGUS. Interestingly, the excess risk of MGUS remained significant with >5 years of latency between the diagnoses of the autoimmune disorder and MGUS, for most conditions. This decreases the possibility of detection bias, where the diagnosis of an autoimmune disease is linked to the work-up of a plasma cell disorder (3). In contrast to several other studies, significant associations between discoid lupus erythematous, autoimmune hemolytic anemia and MGUS were not detected (3, 4, 9). A personal history of autoimmune diseases without detectable autoantibodies was associated with an elevated risk of MM (OR 1.4, 95% CI 1.2-1.6), in addition to a personal history of autoimmune hemolytic anemia, polymyalgia rheumatica and giant cell arteritis (**Table 1**). The study also reported a significantly increased risk of MGUS among patients with a family history of an autoimmune disease (OR 1.1, 95% CI 1.0-1.2), suggesting there might be a shared genetic susceptibility for immune-related conditions and MGUS (3).

In contrast to the abovementioned studies, a population-based study on disease associations with MGUS by Bida et al. did not show significant associations between MGUS and pernicious anemia, systemic sclerosis, ankylosing spondylitis or Sjögren syndrome (**Table 1**) (3, 4, 6). This was the first study estimating such associations based on a cohort specifically screened for MGUS, where 17,398 individuals in Olmsted County, Minnesota, were screened for the disorder (6).

A systematic review by McShane et al. including Brown et al.’s, Lindqvist et al.’s, and Bida et al.’s studies reported a 1.42-fold pooled relative subsequent risk of MGUS in individuals with any autoimmune disorder (RR 1.42, 95% CI 1.14-1.75), and a 1.64-fold risk in those with a previous history of autoimmune disorder with detectable autoantibodies (RR 1.64, 95% CI 1.36-1.97). Autoimmune diseases with systemic and organ involvement were also associated with an increased MGUS risk (RR 1.95 and 1.55, 95% CIs 1.57-2.42 and 1.35-1.79). Polymyositis/dermatomyositis, RA, systemic sclerosis, pernicious anemia, and ankylosing spondylitis were significantly associated with a higher MGUS-risk. No excess risk of MGUS was reported among individuals with autoimmune diseases without detectable antibodies or Sjögren syndrome (**Table 1**) (5).

## MGUS and Infections

Prior history of various infections has been identified as a possible trigger for the pathogenesis of MGUS and MM (4, 34–36). Several epidemiological studies have examined the associations between specific infections and subsequent risk of MGUS. In a prospective study, Andreone et al. reported an 11% prevalence (27/239) of M-proteins in HCV-positive patients with chronic liver disease, compared with only 1% in HCV-negative controls (1/98; p 0.004). 64% (18/28) of individuals with a detectable M-protein were diagnosed with MGUS (37). In a retrospective, observational study on 69 patients with MGUS, 68% of the 57 that were tested for H. pylori showed signs of this infection (38). A high prevalence of MGUS has also been observed in patients infected by human immunodeficiency virus (HIV), compared with the general population (**Table 2**) (39). These findings support the hypothesis of chronic antigen stimulation as a factor in the pathogenesis of MGUS. Alternatively, they may suggest that undetected MGUS or MM may predispose patients to infections.

**Table 2:** Infections associated with MGUS.

Infections	Number of studies showing significant associations	Number of studies showing no associations
Total infections	2 (3, 4)	–
Viral hepatitis C	1 (37)	2 (3, 6)*
Hepatitis	1 (4)	2 (3, 6)*
Helicobacter pylori	1 (38)	1 (3)
HIV	1 (39)	2 (3, 6)*
Influenza	2 (3, 4)	–
Pneumonia	2 (3, 4)	–
Meningitis	1 (3)	1 (4)
Herpes zoster	2 (3, 4)	–
Intestinal infection	1 (3)	–
Gonorrhea	1 (3)	–
Septicemia	2 (3, 4)	–
Pyelonephritis	1 (3)	–
Cystitis	1 (3)	–
Sinusitis	1 (3)	–
Rhinitis	1 (3)	–
Lyme disease	1 (3)	–
Pericarditis	1 (3)	–
Myocarditis	1 (3)	–
Endocarditis	1 (3)	–
Empyema	1 (3)	–
Erysipelas	1 (3)	–
Mycobacterium infections	1 (6)*	–

–indicates not applicable.

*indicates that the study is based on a screened population.

Larger studies have also examined associations between infections and subsequent risk of MGUS. A 1.4-fold risk of MGUS (RR 1.40, 95% CI 1.27-1.53) in individuals with a prior history of infections was detected in a large retrospective study on 4 million black and white, male US veterans, compared with non-MGUS controls. A previous diagnosis of influenza, pneumonia, hepatitis, meningitis, and herpes zoster, >1 year before the diagnosis of MGUS, was also associated with an excess risk of MGUS, suggesting that these conditions might act as triggers for the development of MGUS (**Table 2**). The authors also reported a significantly elevated risk of MM with prior infectious history (RR 1.29, 95% CI 1.20-1.38) (4).

In their population-based study in Sweden, Lindqvist et al. reported a 1.6-fold risk of MGUS in individuals with a prior history of infections overall (OR 1.6, 95% CI 1.5-1.7). A personal history of pneumonia, intestinal infection, gonorrhea, septicemia, herpes zoster, pyelonephritis, cystitis, sinusitis, rhinitis, influenza, meningitis, Lyme disease, pericarditis, myocarditis, endocarditis, empyema, and erysipelas was also associated with a significantly increased risk of MGUS. However, HCV-, H. pylori or HIV-infections were not associated with an increased MGUS-risk (**Table 2**). A prior history of infections was also associated with a significantly increased risk of MM (OR 1.2, 95 CI 1.1-1.3) (3). Bida et al.’s population-based screening study on MGUS-patients in Olmsted County, Minnesota, did not find significant associations between MGUS and HCV infections, concurring with the findings of Lindqvist et al. (3, 6) (**Table 2**). Additionally, previously unreported associations were found between mycobacterium infection and MGUS (6). These results support previous findings that infections might be the trigger for IgH-translocations carried by clonal plasma cells, seen in around 50% of MGUS-patients. These translocations are thought to be of importance for initiation and support of clonal proliferation (40, 41).

## MGUS and Chronic Antigen Stimulation

In the abovementioned studies an increased risk of MGUS has been associated with a previous history of both autoimmune diseases and infections. This supports the theory of chronic antigen stimulation increasing the risk of plasma cell dyscrasias (33). The theory suggests that chronic stimulation by activated immune cells may eventually lead to mutations in actively dividing plasma cells, leading to clonal proliferations and predisposing individuals to increased risk of malignancy (33, 42).

These findings are of course important puzzles in the understanding of the pathogenesis of MGUS and MM but may also be of clinical significance. Although the monoclonal immunoglobulins in MGUS and MM are assumed to be non-functional, recent studies have shown that in a subset of patients, these antibodies target specific infectious antigens. These include Epstein-Barr virus (EBV), HCV, CMV, herpes simplex virus 1- and 2 (HSV-1, HSV-2), varicella zoster virus, H. pylori, Toxoplasma gondii, and Borrelia burgdorferi (38, 43, 44). These results further suggest that chronic antigen stimulation may contribute to the initiation of MGUS and MM and raise the question if treating these pathogens could prevent progression of MGUS to MM. They also introduce the possibility of adding antiviral or antibacterial drugs to current treatment protocols in MM. However, as mentioned above, the risk of MM in individuals with a previous history of autoimmune diseases or infections is considerably lower than the risk of MGUS (3, 4). This might suggest a biological difference of the MGUS found in individuals with a previous history of those conditions compared with those without. This is supported by the findings of Baldursdottir et al, who recently reported a lower risk of progression to MM in MGUS patients with preceding autoimmune diseases, compared with MGUS patients without such disorders (45).

## Methodological Issues: Clinical versus Screened MGUS

Since MGUS is asymptomatic and systematic screening is not recommended, it is typically diagnosed incidentally, during clinical workup for often unrelated medical problems (11). Therefore, it is virtually impossible to determine when exactly MGUS is developed. Furthermore, since MGUS diagnoses are normally incidental, most individuals remain undiagnosed. As a result, most studies are based on clinical cohorts and do not include undiagnosed individuals. This is a common limitation of many MGUS studies, including some of those described in this article, and may have led to selection bias, resulting in generally biased detection of MGUS-patients in their cohorts compared with the general MGUS-population. Therefore, an overestimation of associations between MGUS and various medical problems, including immune-related conditions, is possible. This is supported by the findings of Bida et al. in their population-based screening study on disease associations with MGUS, where only 14 out of the 75 previously reported associations with MGUS were confirmed (6).

### The iStopMM Study

The Iceland Screens, Treats or Prevents Multiple Myeloma (iStopMM) study is the first population-based screening study for MGUS including a randomized trial of follow-up strategies. In 2016-2018, all Icelandic residents born before 1976 were invited to participate in the study. Serum samples were gathered alongside blood sampling in the Icelandic health care system and screened for MGUS by serum protein electrophoresis and FLC assay. Participants diagnosed with MGUS were randomized into three different study arms. In arm 1, participants are not made aware of their MGUS diagnosis and carry on as if the study never took place. Arm 2 follows current guidelines while arm 3 follows a more intensive follow-up strategy. 80,759 individuals (54.3% of the target population) provided informed consent and 75,422 provided serum samples for screening (46). The extensive data collected in the study will help shed a light on important questions on MGUS. By cross-linking the data to high-quality national registries, the study provides a unique opportunity to evaluate the scope of selection bias in previous studies on MGUS by comparing clinically diagnosed MGUS-individuals with those diagnosed by screening. This way, true disease associations with MGUS can be estimated, which will provide important information for increasing our understating of its pathogenesis (47).

## Future Directions

Although current evidence suggests that a prior history of autoimmune diseases and infections increases the risk of both MGUS and MM, further research is needed to determine the impact of these associations. A common limitation to most previous studies on MGUS and its associations with other diseases, is the fact that they are based on clinical cohorts, resulting in selection bias. Therefore, screening studies, such as the iStopMM study, are expected to be pivotal when it comes to evaluation of the true biological- and epidemiological implications of MGUS, before causation is confirmed.

## Author Contributions

AS and SK contributed to the conception and design of the article. AS wrote the first draft of the manuscript. TL and SK administered manuscript revision. All authors read and approved the submitted version.

## Conflict of Interest

The authors declare that the research was conducted in the absence of any commercial or financial relationships that could be construed as a potential conflict of interest.

## Publisher’s Note

All claims expressed in this article are solely those of the authors and do not necessarily represent those of their affiliated organizations, or those of the publisher, the editors and the reviewers. Any product that may be evaluated in this article, or claim that may be made by its manufacturer, is not guaranteed or endorsed by the publisher.
